# Preliminary evidence of effectiveness of preparations from *Nigella sativa* and its leading compound thymoquinone in periodontal therapy

**DOI:** 10.3389/froh.2026.1875959

**Published:** 2026-08-28

**Authors:** Sigrun Chrubasik-Hausmann, Julia Vlachojannis, Marietta Wolf, Fabian Cieplik, Ali Al-Ahmad

**Affiliations:** 1 Faculty of Medicine, Institute of Forensic Medicine, University of Freiburg, Freiburg, Germany; 2 Department of Operative Dentistry and Periodontology, Faculty of Medicine, Medical Center – University of Freiburg, Freiburg, Germany

**Keywords:** adjunctive therapy, black seed, evidence of effectiveness, gingivitis, *Nigella sativa*, oral diseases, peri-implantitis, periodontitis

## Abstract

**Background:**

Preparations of *Nigella sativa* and its major bioactive compound thymoquinone (TQ) possess antioxidant, antimicrobial, anti-inflammatory, and wound-healing properties. These characteristics suggest potential utility as adjuncts in the management of gingivitis, mucositis, periodontitis, and peri-implantitis.

**Objective:**

To critically evaluate the current clinical evidence regarding the use of *Nigella sativa* preparations and TQ in periodontal therapy.

**Methods:**

A systematic literature search following PRISMA guidelines was conducted in PubMed, Embase, Scopus, Web of Science, and Cochrane CENTRAL for English-language clinical studies published through July 2026. Eligible studies investigated preparations of *Nigella sativa* or TQ for periodontal inflammatory diseases. Hand-searching was performed to identify additional relevant publications.

**Results:**

Fourteen exploratory clinical trials were identified. Overall, adjunctive use of *Nigella sativa* preparations or TQ was associated with improvements in clinical periodontal outcome measures. However, interpretation is limited by small sample sizes, methodological heterogeneity, short follow-up periods, and insufficient characterization of the investigational products. Only one of the studies adequately reported the administered daily TQ dose.

**Discussion:**

Without knowledge of the administered TQ dose, neither dose–response relationships nor reliable sample-size calculations for confirmatory trials can be established. Experimental evidence nevertheless supports the biological plausibility of the observed clinical findings and provides a rationale for further clinical investigation.

**Conclusions:**

Current clinical evidence remains preliminary and hypothesis-generating despite encouraging findings. Future confirmatory randomized controlled trials should employ well-characterized, TQ-standardized preparations and report the administered TQ dose to enable reproducibility, dose–response assessment, and evidence-based study planning.

## 1 Introduction

Periodontal diseases are highly prevalent chronic inflammatory conditions associated with systemic disorders, including cardiovascular disease, metabolic syndrome, and neurodegenerative diseases (1–3). Disease initiation is driven by dysbiotic biofilms and a dysregulated host immune response, leading from reversible gingivitis to irreversible tissue destruction in periodontitis (4–6).

While effective oral hygiene prevents gingival inflammation, active periodontitis requires professional subgingival instrumentation and lifelong supportive care  (7, 8). The EFP S3 clinical guideline recommends a stepwise approach including mechanical biofilm control, risk-factor management, subgingival instrumentation, surgical intervention when needed, and supportive maintenance  (7). The endpoint is pocket closure (≤ 4 mm probing depth and no bleeding on probing)  (9).

A central challenge in periodontal therapy lies in the persistence and tissue invasion of periodontitis-associated bacteria supporting biofilm formation (6). Such biofilms show markedly enhanced tolerance—up to 1,000-fold—to antimicrobial agents compared with planktonic bacteria  (10, 11). Consequently, adjunctive antimicrobial strategies have been explored. Systemic antibiotics (typically metronidazole + amoxicillin) are reserved for defined indications due to resistance concerns  (12), while topical antiseptics such as chlorhexidine (CHX) show only modest additional benefit  (13) and can contribute to antimicrobial resistance and dysbiosis  (14, 15).

These limitations have prompted interest in herbal medicines which combine affordability, high biocompatibility, and favorable safety  (16). Among the phytotherapeutics, *Nigella sativa* (black seed) and its leading compound thymoquinone (TQ) display potent antioxidant, antimicrobial, anti-inflammatory, and bone-regenerative actions  (17–20). This systematic review summarizes and critically assesses the evidence for the clinical effectiveness of preparations from *Nigella sativa* in periodontal therapy.

## 2 Methods

Following peer-review, the original PubMed search was expanded to include Embase, Scopus, Web of Science and Cochrane Central Register of Controlled Trials (CENTRAL). The systematic review was subsequently conducted according to the Preferred Reporting Items for Systematic Reviews and Meta-Analyses (21) statement using a rigorous methodology to minimize the risk of bias. Ethical approval was not required because all data were obtained from previously published studies.

The final search of all databases was completed on July 16, 2026; therefore, publications indexed after this date were not eligible for inclusion. The search strategy combined terms related to *Nigella sativa* and its active constituent TQ with terms describing gingivitis, oral mucositis, periodontitis, and peri-implantitis. The following search string was adapted for each database: (“Nigella sativa” OR “black cumin” OR “black cumin seed” OR “black seed” OR kalonji OR thymoquinone) AND (periodontitis OR gingivitis OR “oral mucositis” OR stomatitis OR “periodontal disease*” OR “oral disease*” OR “oral inflammation” OR “oral health”). Eligible studies included randomized controlled trials, randomized clinical trials, controlled clinical trials, and prospective clinical studies published in English and involving human participants. Exclusion criteria comprised *in vitro* studies, animal studies, narrative or systematic reviews, case reports or case series, studies investigating aggressive periodontitis (Grade C periodontitis) or multicomponent interventions in which the independent effect of *Nigella sativa* or TQ could not be determined, and studies outside the predefined disease spectrum of the present review.

Following the initial search, all the retrieved results were imported into Excel for extraction. All articles where a *Nigella sativa* preparation was either used alone or compared to any other intervention for gingivitis/mucositis or peri-implantitis-periodontitis were included. Duplicates were removed before title and abstract screening. Two reviewers (SCH and AA) independently performed title/abstract screening and full-text assessment according to the predefined eligibility criteria. Discrepancies at the full-text stage were resolved through discussion and consultation with a third reviewer (JV). Data were extracted using a customized data extraction form. The PICO framework was used to structure the focus of this literature review, ensuring a comprehensive and systematic approach to evaluating clinical studies where Nigella sativa preparations were used to improve oral health. The PICO components were defined as follows:

P: Patients—The review included patients of all ages with gingivitis/mucositis and peri-implantitis/periodontitis.
I: Intervention—The interventions considered were preparations from Nigella sativa seed.
C: Comparison—Comparisons were made between the groups (placebo or active control).
O: Outcome—The primary outcome was improvement in gingival, mucosal, and periodontal health assessed using established outcome measures appropriate for the condition investigated, including clinical indices (e.g., PI, GI, BOP, PPD, CAL, OMAS, WHO mucositis grade), patient-reported outcomes (e.g., VAS pain and swallowing), and biochemical markers (e.g., IL-6, TNF-α, MMP-8, TAC, ALP). Data were extracted using a customized data extraction form. For the purpose of this review, the following information was extracted and tabulated for all the selected studies: title; first author's name; study design, publication year; study location, population description; intervention, frequency, mg TQ per dose, comparator, established outcome measure(s), follow-up period, and overall RoB. A meta-analysis was not performed due to the significant clinical and methodological heterogeneity between studies.

## 3 Clinical evidence

### 3.1 Overview of search

**Figure 1:**
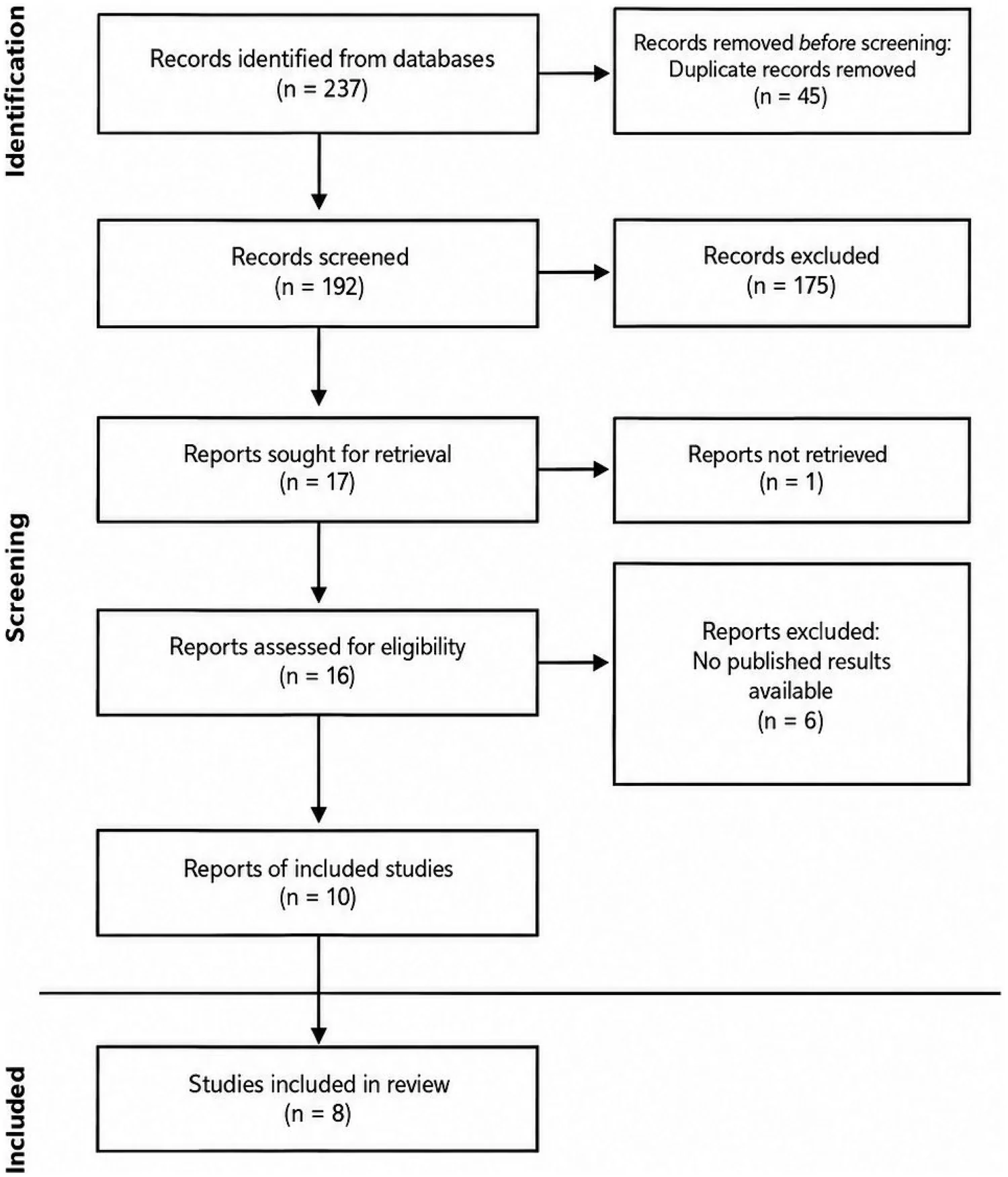
PRISMA 2020 flow diagram of study selection. Records refer to database search results; Reports refer to documents describing a study (e.g., journal articles or trial registry records); Studies refer to unique clinical studies.

Figure 1 presents the PRISMA 2020 flow diagram of the study selection process. The detailed search strategies are presented in Supplementary Table S1. The literature search identified 237 records from PubMed, Embase, Scopus, Web of Science, and the Cochrane Central Register of Controlled Trials (CENTRAL) (see Supplementary Table S2a). After removal of 45 duplicate records (see Supplementary Table S2b), 192 titles and abstracts were screened according to the predefined eligibility criteria. Potentially relevant reports underwent full-text assessment by two independent reviewers. Reports were excluded if they investigated an inappropriate intervention, used an ineligible study design, addressed diseases outside the predefined scope of this review, or if the full text could not be retrieved.

The database searches yielded eight eligible published clinical studies (see Supplementary Table S3). An additional six eligible clinical studies were identified through hand searching, resulting in a total of 14 studies included in the qualitative synthesis (Tables 1, 2).

**Table 1:** Characteristics of the included studies and overall risk of bias assessment (RoB 2) for gingivitis/mucositis.

Author	Arab Farashahi	Singh	Ilangovan	Khan	Almehmadi	Hussain	Ameen
Study design	triple-blind, parallel-group randomized controlled trial	split-mouth clinical trial	double-blind, parallel-group randomized controlled trial	prospective, single-blind, simple randomized controlled trial	double-blind, parallel, randomized controlled trial	open-label controlled trial with two parallel arms	prospective open-label clinical trial
Year	2026	2019	2021	2021	2024	2021	2019
Country	Iran	India	India	USA	Saudi Arabia	Iraq	Iraq
Participants	36 patients with gingivitis (19 F, 17 M)	24 patients with moderate-to-severe gingivitis (25–45 years; NR)	30 participants with gingivitis (18–28 years; NR)	60 children with gingivitis (12–18 years; NR)	63 participants with gingivitis (18–40 years; NR)	52 patients with chemotherapy-induced oral mucositis (<25 to >45 years; 27 M, 25 F)	40 patients with chemotherapy-induced oral mucositis (28 M, 12 F)
Intervention	20% Nigella sativa mouthwash, 10 mL	Nigella sativa ethanolic extract (1:3, 1:1, 3:1 in glycerin), 1 mL	20% Nigella sativa mouthwash, 10 mL	0.1% Nigella sativa gel (pea-sized amount)	Standard care + 5% Nigella sativa gel	Commercial Nigella sativa oil mouthwash, 10 mL	Commercial Nigella sativa oil mouthwash, 10 mL
Frequency	twice daily	repeated for 3 consecutive days	twice daily	twice daily	twice daily	four times daily	five times daily
mg TQ/Dose	NR	NR	NR	NR	NR	NR	NR
Comparator	CHX 0.2% mothwash	SRP alone	CHX 0.12% mothwash	CHX 0.12% gel	SC + placebo gel, SC alone	moutwash^a^	moutwash^a^
Outcome	significant reductions in PI and PBI	significant reductions in PI and GI	significant reductions in PI and GI	significant reductions in PI and GI	significant reductions in PI and PBI	significant reductions in OMAS, VAS pain, VAS swallowing and salivary IL-6	significant reductions in VRS mucositis severity and salivary Il-6 + TNFa
Follow-Up	2 weeks	28 days	2 weeks	28 days	2 weeks	28 days	60 days
Overall RoB	some concerns	some concerns	some concerns	low risk	low risk	some concerns	some concerns

Ns, *Nigella sativa*; SRP, scaling and root planing; SC, standard protocol of scaling; PI, plaque index; PBI, papillary bleeding index; GI, gingival index; OMAS, oral mucositis assessment scale; VAS, visual analogue scale, IL-6 interleukin-6, TNF-a tumor necrosis factor-a, NR not reported.

a nystatin 1,00,000 U tetracycline 0.02% lidocaine 0.5%, dexamethasone 0.5%.

**Table 2:** Characteristics of the included studies and overall risk of bias assessment (RoB 2) for peri-implantitis/ periodontitis.

Author	Thanasekaran	Kapil	Al-Bayaty	Khalil	Alaaeldin	Hassan	Nedumaran
Study design	prospective randomized controlled trial	prospective, randomized controlled trial	andomized, single-blind split-mouth clinical trial	prospective, controlled clinical trial	prospective, controlled clinical trial	triple-blind, parallel-arm, randomized controlled trial,	prospective, controlled clinical study
Year	2025	2018	2013	2019	2017	2020	2026
Country	India	India	Malaysia	Egypt	Egypt	Pakistan	India
Participants	40 patients with peri-implantitis (25–60 years; NR)	20 patients (40 periodontal sites) with chronic periodontitis (23–61 years; 15 M, 5 F)	12 M with chronic periodontitis (180 periodontal pockets ≥5 mm)	48 patients with moderate-to-severe chronic periodontitis (25–58 years) and 20 healthy controls	48 patients with moderate-to-severe chronic periodontitis (25–58 years) and 20 healthy controls	50 patients with chronic periodontitis (40 completed; NR)	30 patients with Stage II Grade A periodontitis (20–55 years; NR)
Intervention	mechanical debridement + 0.2% TQ gel (single subgingival application)	SRP + 0.2% TQ gel (subgingival application)	SRP + TQ biodegradable chitosan chip (subgingival insertion)	SRP + 0.1% TQ chitosan gel (subgingival application)	SRP + 0.1% TQ carbopol-poloxamer gel (subgingival application)	SRP + Nigella sativa oil mouthwash	SRP + single application of 0.2% TQ gel
Frequency	single application at baseline	four applications (baseline, weeks 2, 3 and 4)	two chip insertions (baseline and day 14)	two applications (immediately after SRP and after 48 h)	two applications (immediately after SRP and after 48 h)	twice daily rinses	single application at baseline
TQ/Dose	NR	NR	0.25 mg/chip	NR	NR	NR	NR
Comparator	mechanical debridement alone	SRP alone	SRP alone (control group), SRP + chitosan chip	SRP alone	SRP alone	SRP + normal saline	SRP alone
Outcomes	significant within group improvements in PI, GI, PPD and CAL after 3 months in both groups, with greater improvements in the TQ group	significant reductions in PPD, CAL and GCF-ALP levels compared with SRP alone; no significant intergroup differences for PI and GI; no adverse effects reported	significant improvements in PI, BOP, and PPD and CAL with greater improvements in the TQ group compared with the other groups	significant within-group improvements in PI, GI, PPD and CAL at 4 and 12 weeks. Statistical between-group comparison and additional clinical benefit of TQ gel over SRP alone unclear	significant improvements in PI, GI, PPD, CAL, reduced IL-1β levels, and increased TAOC levels in both groups, with superior improvements in the TQ group.	salivary MMP-8 levels decreased in both groups; adjunctive Nigella sativa mouthwash did not provide additional clinical benefit compared with saline.	compared to the control group, GI, PPD, and CAL were significantly more improved after 3 months, as were salivary TOS, TAOC and OSI
Follow-up	3 months	6 weeks	60 days	12 weeks	12 weeks	2 weeks	3 months
Overall RoB	some concerns	some concerns	low risk	high risk	high risk	low risk	high risk

SRP, scaling and root planing; PPD, peri-implant/pocket probing depth; CAL, clinical attachment level; GCF, gingival crevicular fluid; ALP, alkaline phosphatase; OP, bleeding upon probing; TAOC, total antioxidant capacity; TOS, total oxidant status; OSI, oxidative stress index; TQ, thymoquinone; NR, not reported.

The CENTRAL search required particular consideration because it indexed both published articles and trial registry records. Twenty-five clinical records were identified, of which 18 were considered potentially relevant. These comprised five published full-text articles included in the review and 13 trial registry records referring to completed and published, ongoing, completed but unpublished, or otherwise unavailable studies. Seven records did not meet the predefined eligibility criteria. Registry records without an eligible published full-text article available by the final search date were not included in the qualitative synthesis.

### 3.2 In gingivitis/mucositis

The characteristics of the included clinical studies are summarized in Table 1. Detailed domain-specific RoB 2 assessments and the rationale for the overall judgements are presented in Supplementary Tables S4, S5.

In terms of study design, one of the five gingivitis studies was a triple-blind (22) and one (23) a double-blind, parallel-group randomized controlled trial. One study had a split-mouth design (24) and two studies were randomized (25, 26), one of them single-blind (25) (Table 1). All studies were exploratory and none was explicitly designed as a confirmatory efficacy trial. The two mucositis studies (27, 28) were open-label studies. Thus, considerable heterogeneity was observed in the study design, but also in the *Nigella* formulations, TQ concentrations, dosages, frequency of application and treatment duration. Four studies used a *Nigella* mouthwash, two a gel formulation that differed in their galenic and one study used an ethanolic extract in three dilutions with glycerin. The daily dose of TQ has not been reported in any of the studies. The overall risk of bias was some concerns in five of the seven studies, only two studies had a low risk of bias. Detailed domain-specific RoB 2 assessments and their justifications are presented in Supplementary Tables S4, S5. Overall, the evidence for the effectiveness of Nigella sativa preparations in gingivitis and oral mucositis remains limited.

### 3.3 In peri-implantitis/periodontitis

The study characteristics are summarized in Table 2. Detailed domain-specific RoB 2 assessments and the rationale for the overall judgements are presented in Supplementary Tables S4, S5. One prospective randomized controlled clinical trial investigated patients suffering from peri-implantitis (29). Four of the periodontitis studies were prospective controlled clinical trials (30–33) of which only one was randomized (30). One study had a randomized single-blind split-mouth clinical trial design (34) and one was a parallel-arm randomized controlled, triple-blinded trial (35). All studies were exploratory, none was adequately powered for efficacy confirmation. Considerable heterogeneity was observed in the study design, but also in the intervention, the frequency of application, the *Nigella sativa* preparation, comparator and evaluation period. Five studies used a gel with 0.2% (29, 30, 33) or 0.1% (31, 32) of TQ. The galenic gel formulations were, however, not identical. One study inserted chips at baseline and day 14, each containing 0.25 mg of TQ (34) and one study used an uncharacterized *Nigella sativa* oil mouthwash (35) (Table 2). The daily TQ dose was reported in only one study.

The overall risk of bias in the peri-implantitis study was some concerns. A low risk of bias was found in only two of the periodontitis studies whereas it was worse in the others. Detailed domain-specific RoB 2 assessments and their justifications are presented in Supplementary Tables S4, S5. The evidence of effectiveness of preparations of *N. sativa* or its leading compound TQ in the treatment of peri-implantitis or periodontitis is currently limited.

## 4 Discussion

### 4.1 Clinical evidence current status

Clinical evidence for gingivitis/mucositis is limited to small exploratory trials investigating *N. sativa* mouthwashes, extract and gels. Across studies, improvements in plaque and gingival indices were observed, with some formulations demonstrating comparable or occasionally superior effects to chlorhexidine in short-term applications (22–28). However, these findings must be interpreted cautiously. Most earlier studies were characterized by methodological limitations including inadequate reporting of randomization procedures and lack of prospective sample-size calculations. In contrast, the recent trial by Arab Farashahi et al. addressed several of these methodological shortcomings through prospective registration, triple blinding, allocation concealment, intention-to-treat analysis, and an *a priori* sample-size calculation. Nevertheless, the short follow-up and exploratory sample size still limit definitive conclusions. Overall, the available evidence suggests potential short-term benefits but remains insufficient for clinical recommendations.

In the two gingivitis studies (22, 23) and the two mucositis studies (27, 28), *Nigella sativa* mouthwashes were used; however, none of the studies reported the TQ content in the daily administered dose. Although one group states that the extract was standardized to approximately 1.5% TQ, the analytical method described (Folin–Ciocalteu assay) determines total phenolic content rather than TQ specifically which is best determined by HPLC. Consequently, the reported TQ standardization cannot be independently verified and the actual TQ content of the administered mouthwash remains uncertain. Stating that the mouthwash was prepared from a 20% aqueous extract is insufficient to ensure reproducibility of the study, as the TQ content of *Nigella sativa* seeds can vary considerably (36), as can the TQ concentration in Ns oils, which we have previously demonstrated (37). The addition of honey and peppermint extract as flavouring agents may have introduced biological confounding, as both possess documented anti-inflammatory and antimicrobial activities (38, 39). The two mucositis studies undertaken by the same group used a commercial mouthwash preparation; however, the manufacturer's website could not be accessed (“Bandwidth Limit Exceeded”), preventing verification of its composition. However, reporting the TQ content of the administered daily dose is essential for planning adequately powered confirmatory studies. Without knowledge of the administered TQ dose, neither dose-response relationships nor clinically meaningful sample size calculations can be established. The remaining three gingivitis studies investigated different gel formulations: a 5% TQ gel incorporated into a novel liposomal drug delivery system (26), an ethanolic *N. sativa* extract formulated with varying glycerin concentrations (24), and a lipid-based gel containing 0.1% TQ (25). None of these studies specified the amount of TQ delivered per application or per daily dose, limiting reproducibility and comparison across studies. Merely stating that a pea-sized amount (25) or 1 mL (24) of gel was administered is not enough. An inconsistency was noted in the publication by Almehmadi et al. (26). The intervention was described as a “5% TQ gel”; however, the formulation details indicate a final concentration of 0.2% (20 mg TQ in 10 g gel). Consequently, the actual TQ concentration used in the study is internally inconsistent and the TQ dose per application remains unknown. Consequently, none of these studies provides an adequate basis for planning a confirmatory randomized controlled trial.

A limited number of exploratory clinical trials have investigated TQ as an adjunct to non-surgical periodontal therapy in the treatment of peri-implantitis/ periodontitis (29–35). These studies generally report improvements in clinical parameters, including probing depth, plaque index, gingival index, and clinical attachment level. Despite consistent directional trends, considerable heterogeneity exists regarding study design, formulation, dosing regimens, and outcome assessment. For example, three periodontitis studies evaluated a gel containing 0.2% TQ in carbopol (29, 30, 33) but did not report the amount of TQ applied per dose. Khalil investigated a TQ-loaded chitosan gel, Al-Bayaty used TQ-containing chitosan chips. Whereas Khalil did not provide the TQ content per gel application, the chips used by the Al-Bayaty group contained 0.25 mg TQ. This is the only study that provides sufficient information to permit independent replication. An important limitation of the study by Hassan et al. is the lack of characterization of the *Nigella sativa* oil. Neither TQ concentration nor any standardization or phytochemical analysis was reported. Since commercially available Nigella sativa oils may differ considerably in their TQ content (37), the administered dose of the presumed active constituent remains unknown, which limits interpretation and comparison with other studies**.**

Because the study medications were insufficiently characterized, six of the seven peri-implantitis/periodontitis trials cannot serve as an adequate basis for planning a confirmatory trial. The only trial using chips with 0.25 mg TQ can be considered a pilot study (34). Although no *a priori* sample size calculation was reported, a retrospective estimation suggests that the study was likely adequately powered to detect the relatively large difference observed between the TQ chip and SRP alone. However, it was probably underpowered to detect the smaller difference between the TQ and chitosan chips. Based on the observed CAL difference of 0.51 mm between the TQ chip and SRP alone, approximately 13 and 17 evaluable patients would have been required to achieve 80% and 90% power, respectively, assuming a standard deviation of the paired differences of 0.60 mm (two-sided *α* = 0.05). As the variance of the paired differences was not reported, these estimates should be interpreted with caution and no firm conclusions regarding sample-size requirements can be drawn. Thus, clinical evidence for peri-implantitis/periodontitis remains preliminary and does not allow firm conclusions regarding efficacy.

Taken together, based on current evidence, preparations of *N. sativa* or TQ cannot be recommended as replacements for established therapies of gingivitis, mucositis, peri-implantitis and periodontitis. Their use should currently be considered experimental, with potential as adjunctive agents pending further clinical validation.

### 4.2 Experimental and mechanistic evidence

A substantial body of preclinical research supports the biological plausibility of *N. sativa* and TQ in periodontal therapy.

#### 4.2.1 Anti-inflammatory and wound-healing effects

Animal studies demonstrate that *N. sativa* and TQ reduce pro-inflammatory mediators, including TNF-α, PGE-2, and matrix metalloproteinases, in animal models of oral inflammation and mucosal injury (40–46). Additionally, enhanced epithelialization, reduced oxidative stress, and improved tissue repair have been reported.

#### 4.2.2 Antimicrobial and antibiofilm activity

TQ exhibits inhibitory effects against key periodontal pathogens, including *Porphyromonas gingivalis* and *Fusobacterium nucleatum*, and significantly reduces biofilm formation *in vitro* (47–49). Broad-spectrum antimicrobial activity has been demonstrated against both bacterial and fungal species relevant to oral health (50, 51).

#### 4.2.3 Modulation of microbial interactions and resistance mechanisms

Experimental evidence suggests that TQ interferes with microbial virulence factors and polymicrobial interactions, including bacterial–fungal synergism (52). Additional studies indicate potential antimicrobial resistance-modulating effects, including plasmid curing and restoration of antibiotic sensitivity (53).

#### 4.2.4 Effects on bone metabolism

Preclinical studies further suggest that TQ may reduce alveolar bone loss in experimental periodontitis models, indicating potential modulation of host tissue responses (54–56).

## 5 Conclusions

The current evidence for the clinical efficacy of *Nigella sativa* and TQ in gingival and periodontal therapy remains insufficient despite encouraging preliminary findings. Experimental data consistently support plausible anti-inflammatory, antimicrobial, and regenerative effects.

Future confirmatory randomized controlled trials are needed to determine effect sizes, optimize TQ-standardized formulations, and assess long-term adjunctive utility in gingivitis, mucositis, peri-implantitis and periodontitis.
