# Supplementary material for: Preliminary evidence of effectiveness of preparations from Nigella sativa and its leading compound thymoquinone in periodontal therapy
Source: Front Oral Health. 2026 Aug 28;7:1875959. doi: 10.3389/froh.2026.1875959 (PMC13563123; doi:10.3389/froh.2026.1875959)
Supplement: Supplementary file 1 [file Table1.docx]

**Supplementary Material**

**Supplementary Table S1: Detailed search strategies for the five electronic databases.**

The search strategy was adapted to the indexing system and syntax of each database while maintaining the same search concepts across all databases.
**Intervention terms:** "Nigella sativa" OR "black cumin" OR "black seed" OR kalonji OR thymoquinone  **Disease terms:** periodontitis OR gingivitis OR peri-implantitis OR oral mucositis OR oral inflammation

Searches were completed on July 16, 2026.

| **Database** | **Search period** | **Controlled Vocabulary** | **Free Text Terms** | **Search string** | **Filters applied** | **Records retrieved** |
| --- | --- | --- | --- | --- | --- | --- |
| PubMed | Database inception to July 2026 | MeSH | Yes | ("Nigella sativa" OR "black cumin" OR "black seed" OR kalonji OR thymoquinone) AND (periodontitis OR gingivitis OR oral mucositis OR peri-implantitis OR oral inflammation) | Humans, English | 38 |
| Embase | Database inception to July 2026 | Emtree | Yes | ('nigella sativa'/exp OR 'Nigella sativa':ti,ab OR 'black seed':ti,ab OR 'black cumin':ti,ab OR 'black cumin seed':ti,ab OR kalonji:ti,ab OR thymoquinone:ti,ab) AND ('periodontitis'/exp OR 'gingivitis'/exp OR 'mucositis'/exp OR periodontitis:ti,ab OR gingivitis:ti,ab OR mucositis:ti,ab OR 'oral mucositis':ti,ab OR stomatitis:ti,ab OR 'periodontal disease*':ti,ab OR 'oral disease*':ti,ab OR 'oral inflammation':ti,ab OR 'oral health':ti,ab) | English | 70 |
| Scopus | Database inception to July 2026 | Not applicable | Yes | TITLE-ABS-KEY ("Nigella sativa" OR "black seed" OR "black cumin" OR "black cumin seed" OR kalonji OR thymoquinone) AND TITLE-ABS-KEY (periodontitis OR gingivitis OR mucositis OR "oral mucositis" OR stomatitis OR "periodontal disease*" OR "oral disease*" OR "oral inflammation" OR "oral health") | English | 2 |
| Web of Science | Database inception to July 2026 | Not applicable | Topic Search | TS=("Nigella sativa" OR "black seed" OR "black cumin" OR "black cumin seed" OR kalonji OR thymoquinone) AND TS=(periodontitis OR gingivitis OR mucositis OR "oral mucositis" OR stomatitis OR "periodontal disease*" OR "oral disease*" OR "oral inflammation" OR "oral health") | English | 102 |
| Cochrane Central | Database inception to July 2026 | Not applicable | Yes | ("Nigella sativa" OR "black seed" OR "black cumin" OR "black cumin seed" OR kalonji OR thymoquinone) AND (periodontitis OR gingivitis OR mucositis OR "oral mucositis" OR stomatitis OR "periodontal disease*" OR "oral disease*" OR "oral inflammation" OR "oral health") | English | 25 |

**Supplementary Table S2a. Study identification and classification of records retrieved from the five databases before deduplication**

| No. | Study | Clinical | Animal | In vitro | Review | Other | Potentially eligible | Include in Review | Full text required | Notes | Deduplication Notes | Database |
| --- | --- | --- | --- | --- | --- | --- | --- | --- | --- | --- | --- | --- |
| 1 | Mekhemar |  |  |  | x |  |  | No |  |  |  | PubMed |
| 2 | Al-Attass |  |  |  | x |  |  | No |  |  |  | PubMed |
| 3 | Tada |  |  | x |  |  |  | No |  |  |  | PubMed |
| 4 | Madani |  | x |  |  |  |  | No |  |  |  | PubMed |
| 5 | Singh | x |  |  |  |  |  | Yes |  | Gingivitis |  | PubMed |
| 6 | Setiawatie |  | x |  |  |  |  | No |  |  |  | PubMed |
| 7 | Tawfig, |  |  | x |  |  |  | No |  |  |  | PubMed |
| 8 | Alicea |  |  |  | x |  |  | No |  |  |  | PubMed |
| 9 | Tantivitayakul |  |  | x |  |  |  | No |  |  |  | PubMed |
| 10 | Hussain | x |  |  |  |  |  | Yes |  | Mucositis |  | PubMed |
| 11 | Kaypetch |  |  | x |  |  |  | No |  |  |  | PubMed |
| 12 | Bhavikatti |  |  | x |  |  |  | No |  |  |  | PubMed |
| 13 | Varela-López |  |  |  | x |  |  | No |  |  |  | PubMed |
| 14 | Khan |  |  |  | x |  |  | No |  |  |  | PubMed |
| 15 | Sultan |  |  | x |  |  |  | No |  |  |  | PubMed |
| 16 | Ozdemir |  | x |  |  |  |  | No |  |  |  | PubMed |
| 17 | Afşar |  | x |  |  |  |  | No |  |  |  | PubMed |
| 18 | Nazemi Salman |  |  | x |  |  |  | No |  |  |  | PubMed |
| 19 | Maduratna |  | x |  |  |  |  | No |  |  |  | PubMed |
| 20 | Huang |  |  |  | x |  |  | No |  |  |  | PubMed |
| 21 | Arab Farashahi | x |  |  |  |  |  | Yes |  | Gingivitis |  | PubMed |
| 22 | Sthanislas |  |  | x |  |  |  | No |  |  |  | PubMed |
| 23 | Eğilmez |  | x |  |  |  |  | No |  |  |  | PubMed |
| 24 | Thummuri |  |  | x |  |  |  | No |  |  |  | PubMed |
| 25 | Ariamanesh |  |  | x |  |  |  | No |  |  |  | PubMed |
| 26 | Vlachojannis |  |  | x |  |  |  | No |  |  |  | PubMed |
| 27 | Laskar |  |  | x |  |  |  | No |  |  |  | PubMed |
| 28 | Kiari |  |  | x |  |  |  | No |  |  |  | PubMed |
| 29 | Acipinar |  | x |  |  |  |  | No |  |  |  | PubMed |
| 30 | Maraghehpour |  |  | x |  |  |  | No |  |  |  | PubMed |
| 31 | Al-Khalifa |  |  | x |  |  |  | No |  |  |  | PubMed |
| 32 | Tariq |  |  |  |  | x |  | No |  | mixture |  | PubMed |
| 33 | Almehmadi | x |  |  |  |  |  | Yes |  | Gingivitis |  | PubMed |
| 34 | Xu |  |  | x |  |  |  | No |  |  |  | PubMed |
| 35 | Çanakci |  | x |  |  |  |  | No |  |  |  | PubMed |
| 36 | Kapil | x |  |  |  |  |  | Yes |  | Periodontitis |  | PubMed |
| 37 | Al-Thobity |  | x |  |  |  |  | No |  |  |  | PubMed |
| 38 | Konstantinidis, |  |  |  | x |  |  | No |  |  |  | PubMed |
| 1 | Alicea |  |  |  | x |  |  | No |  |  |  | Embase |
| 2 | Ganeshkumar |  |  | x |  |  |  | No |  |  |  | Embase |
| 3 | Anonymous | x |  |  |  |  |  | No |  | Results not available in VClinicalTrials.gov |  | Embase |
| 4 | Anonymous | x |  |  |  |  |  | No |  | Protocol |  | Embase |
| 5 | Anonymous | x |  |  |  |  |  | No |  | Radiation-induced Dermatitis |  | Embase |
| 6 | Anonymous |  |  |  |  | x |  | No |  | aggressive periodontitis |  | Embase |
| 7 | Anonymous | x |  |  |  |  |  | No |  | registry record corresponding to Rahman et al. 2023 |  | Embase |
| 8 | Anonymous | x |  |  |  |  |  | No |  | registry record; duplicate of the Almehmadi publication. |  | Embase |
| 9 | Anonymous |  |  |  |  | x |  | No |  | no clinical study |  | Embase |
| 10 | Anonymous | x |  |  |  |  |  | No |  | Gastritis |  | Embase |
| 11 | Khan |  |  | x |  |  |  | No |  |  |  | Embase |
| 12 |  |  |  |  |  |  |  |  |  |  | identical publication indexed in PubMed (Record 29) | Embase |
| 13 | Afsar |  | x |  |  |  |  | No |  |  |  | Embase |
| 14 |  |  |  |  |  |  |  |  |  | Gingivitis | identical publication indexed in PubMed (Record 21) | Embase |
| 15 | Thanasekaran | x |  |  |  |  |  | Yes |  | Peri-implantitis |  | Embase |
| 16 | Bhavikatti |  |  | x |  |  |  | No |  |  |  | Embase |
| 17 | Tungare |  |  |  | x |  |  | No |  |  |  | Embase |
| 18 | Ghasemi |  |  |  | x |  |  | No |  |  |  | Embase |
| 19 | Ahmad |  |  |  | x |  |  | No |  |  |  | Embase |
| 20 | Bhavikatti |  |  |  | x |  |  | No |  |  |  | Embase |
| 21 | Joshi |  |  |  | x |  |  | No |  |  |  | Embase |
| 22 | Belgaumi |  |  | x |  |  |  | No |  |  |  | Embase |
| 23 | Madani |  | x |  |  |  |  | No |  |  |  | Embase |
| 24 | Bakhtiari |  | x |  |  |  |  | No |  |  |  | Embase |
| 25 | Tawfig |  |  | x |  |  |  | No |  |  |  | Embase |
| 26 | Rahman | x |  |  |  |  |  | No |  | no results available - ClinicalTrials.gov |  | Embase |
| 27 | Qutieshat |  |  |  | x |  |  | No |  |  |  | Embase |
| 28 | Orororo |  | x |  |  |  |  | No |  |  |  | Embase |
| 29 | Ulfah |  |  | x |  |  |  | No |  |  |  | Embase |
| 30 | Lima |  |  |  | x |  |  | No |  |  |  | Embase |
| 31 | Dilber |  | x |  |  |  |  | No |  |  |  | Embase |
| 32 | Oliveira |  |  | x |  |  |  | No |  |  |  | Embase |
| 33 | Servi |  |  | x |  |  |  | No |  |  |  | Embase |
| 34 | Marmitt |  |  |  | x |  |  | No |  |  |  | Embase |
| 35 | Kazemi |  | x |  |  |  |  | No |  |  |  | Embase |
| 36 | Ahuja |  |  |  | x |  |  | No |  |  |  | Embase |
| 37 | Sultan |  |  | x |  |  |  | No |  |  |  | Embase |
| 38 |  |  |  |  |  |  |  |  |  |  | identical publication indexed in PubMed (Record 6) | Embase |
| 39 | Asri |  |  |  | x |  |  | No |  |  |  | Embase |
| 40 |  |  |  |  |  |  |  |  |  |  | identical publication indexed in PubMed (Record 31) | Embase |
| 41 | Kurnia |  |  | x |  |  |  | No |  |  |  | Embase |
| 42 | Xu |  |  | x |  |  |  | No |  |  |  | Embase |
| 43 |  |  |  |  |  |  |  |  |  |  | identical publication indexed in PubMed (Record 3) | Embase |
| 44 | Tantivitayakul |  |  | x |  |  |  | No |  |  | identical publication indexed in PubMed (Record 9) | Embase |
| 45 | Alwafi |  |  |  |  | x |  | No |  | Epidemiological study |  | Embase |
| 46 | Tsukatani |  |  | x |  |  |  | No |  |  |  | Embase |
| 47 | Anonymous |  |  |  |  | x |  | No |  | Ni clinical study |  | Embase |
| 48 | Alhiyari |  | x |  |  |  |  | No |  |  |  | Embase |
| 49 |  |  |  |  |  |  |  |  |  | Mucositis | identical publication indexed in PubMed (Record 10) | Embase |
| 50 | Ameen | x |  |  |  |  |  | Yes |  | Mucositis |  | Embase |
| 51 | Cheraghi |  |  |  | x |  |  | No |  |  |  | Embase |
| 52 |  |  |  |  |  |  |  |  |  | Periodontitis | Duplicate of PubMed Record 36 | Embase |
| 53 |  |  |  |  |  |  |  |  |  |  | identical publication indexed in PubMed (Record 26) | Embase |
| 54 | Moayer |  | x |  |  |  |  | No |  |  |  | Embase |
| 55 | Elamrousy | x |  |  |  |  |  | No |  | No full-text retrieval was pursued because studies on Grade C periodontitis were excluded a priori according to the predefined eligibility criteria. |  | Embase |
| 56 | Canakci |  | x |  |  |  |  | No |  |  |  | Embase |
| 57 |  |  |  |  |  |  |  |  |  |  | identical publication indexed in PubMed (Record 27) | Embase |
| 58 | Al-Thobity |  |  | x |  |  |  | No |  |  |  | Embase |
| 59 | Naeini |  |  | x |  |  |  | No |  |  |  | Embase |
| 60 | El-Sheikh |  | x |  |  |  |  | No |  |  |  | Embase |
| 61 | Uzun |  |  |  |  |  | x | No |  | Epidemiological study |  | Embase |
| 62 |  |  |  |  |  |  |  |  |  |  | identical publication indexed in PubMed (Record 2) | Embase |
| 63 | Mahdizadeh |  |  |  | x |  |  | No |  |  |  | Embase |
| 64 | Kafar |  |  |  |  |  | x | No |  | Epidemiological study |  | Embase |
| 65 | Guler |  |  |  | x |  |  | No |  |  |  | Embase |
| 66 |  |  |  |  |  |  |  |  |  |  | identical publication indexed in PubMed (Record 24) | Embase |
| 67 | Farrag |  | x |  |  |  |  | No |  |  |  | Embase |
| 68 | Osman |  |  |  |  |  | x | No |  | Coeliac Disease, Case Report |  | Embase |
| 69 |  |  |  |  |  |  |  |  |  |  | identical publication indexed in PubMed (Record 16) | Embase |
| 70 | Kokoska |  |  | x |  |  |  | No |  |  |  | Embase |
| 1 |  |  |  |  |  |  |  |  |  |  | identical publication indexed in PubMed (Record 2) | Scopus |
| 2 | Bhavikatti |  |  | x |  |  |  | No |  |  |  | Scopus |
| 1 | Kokoska, |  |  | x |  |  |  | No |  |  |  | Web of Science |
| 2 |  |  |  |  |  |  |  |  |  |  | identical publication indexed in PubMed (Record 1) | Web of Science |
| 3 | Afsar |  | x |  |  |  |  | No |  |  |  | Web of Science |
| 4 |  |  |  |  |  |  |  |  |  |  | identical publication indexed in PubMed (Record 16) | Web of Science |
| 5 |  |  |  |  |  |  |  |  |  |  | identical publication indexed in PubMed (Record 18) | Web of Science |
| 6 | Moayer |  | x |  |  |  |  | No |  |  |  | Web of Science |
| 7 | Varela-Lopez |  |  |  | x |  |  | No |  |  |  | Web of Science |
| 8 | Supandi |  | x |  |  |  |  | No |  |  |  | Web of Science |
| 9 | Sultan |  |  | x |  |  |  | No |  |  |  | Web of Science |
| 10 |  |  |  |  |  |  |  |  |  |  | identical publication indexed in PubMed (Record 2) | Web of Science |
| 11 | Anonymous, Khan |  |  |  |  | x |  | No |  | mixed product |  | Web of Science |
| 12 | Alhiyari |  |  |  |  | x |  | No |  | Xerostoma |  | Web of Science |
| 13 |  |  |  |  |  |  |  |  |  |  | identical publication indexed in PubMed (Record 26) | Web of Science |
| 14 |  |  |  |  |  |  |  |  |  |  | identical publication indexed in PubMed (Record 19) | Web of Science |
| 15 | Alamoudi |  |  | x |  |  |  | No |  |  |  | Web of Science |
| 16 | Rahman | x |  |  |  |  | x | No |  | Gingivitis |  | Web of Science |
| 17 | Kang |  |  | x |  |  |  | No |  |  |  | Web of Science |
| 18 |  |  |  |  |  |  |  |  |  |  | identical publication indexed in PubMed (Record 31) | Web of Science |
| 19 | Al-Wafi |  | x |  |  |  |  | No |  |  |  | Web of Science |
| 20 | Khan |  |  |  | x |  |  | No |  |  |  | Web of Science |
| 21 | Dirjomuljono |  |  |  |  |  |  | No |  | other plant |  | Web of Science |
| 22 | Dilber |  | x |  |  |  |  | No |  |  |  | Web of Science |
| 23 | Ren |  |  | x |  |  |  | No |  |  |  | Web of Science |
| 24 | Anonymous |  |  | x |  |  |  | No |  |  |  | Web of Science |
| 25 |  |  |  |  |  |  |  |  |  |  | identical publication indexed in PubMed (Record 28) | Web of Science |
| 26 | [Anonymous] |  |  | x |  |  |  | No |  |  |  | Web of Science |
| 27 | Srinivasan |  |  |  | x |  |  | No |  |  |  | Web of Science |
| 28 | Tabassum |  |  |  | x |  |  | No |  |  |  | Web of Science |
| 29 | Kazemi |  | x |  |  |  |  | No |  |  |  | Web of Science |
| 30 | Kurnia |  |  |  | x |  |  | No |  |  |  | Web of Science |
| 31 | Zhang |  |  | x |  |  |  | No |  | Rosmarinic acid |  | Web of Science |
| 32 |  |  |  |  |  |  |  |  |  | Gingivitis | identical publication indexed in PubMed (Record 5) | Web of Science |
| 33 | Abdelfadil |  |  | x |  |  |  | No |  |  |  | Web of Science |
| 34 | Rajkamal |  | x |  |  |  |  | No |  |  |  | Web of Science |
| 35 | Hussein |  |  | x |  |  |  | No |  |  |  | Web of Science |
| 36 | Alaufi |  |  | x |  |  |  | No |  |  |  | Web of Science |
| 37 | Tucakov |  |  | x |  |  |  | No |  |  |  | Web of Science |
| 38 | Sallal, |  |  | x |  |  |  | No |  |  |  | Web of Science |
| 39 | Sanpinit |  | x |  |  |  |  | No |  |  |  | Web of Science |
| 40 | Fath |  |  | x |  |  |  | No |  |  |  | Web of Science |
| 41 | Madani |  | x |  |  |  |  | No |  |  |  | Web of Science |
| 42 | Jo |  |  | x |  |  |  | No |  |  |  | Web of Science |
| 43 | Erdogan |  | x |  |  |  |  | No |  | other plant |  | Web of Science |
| 44 | Imran |  |  |  | x |  |  | No |  |  |  | Web of Science |
| 45 | Pu |  | x |  |  |  |  | No |  |  |  | Web of Science |
| 46 | Khan |  |  |  | x |  |  | No |  | dry sockets after tooth extraction |  | Web of Science |
| 47 |  |  |  |  |  |  |  |  |  | Mucositis | identical publication indexed in PubMed (Record 10) | Web of Science |
| 48 | Siriwattanasatorn |  |  | x |  |  |  | No |  |  |  | Web of Science |
| 49 | Pipalia | x |  |  |  |  |  | No |  | submucous fibrosis |  | Web of Science |
| 50 | Besra |  |  | x |  |  |  | No |  |  |  | Web of Science |
| 51 | Dirjomuljono | x |  |  |  |  |  | No |  | mixture |  | Web of Science |
| 52 | Bhavikatti |  |  | x |  |  |  | No |  |  |  | Web of Science |
| 53 | Almatroodi |  |  |  | x |  |  | No |  | cancer |  | Web of Science |
| 54 |  |  |  |  |  |  |  |  |  |  | identical publication indexed in PubMed (Record 27) | Web of Science |
| 55 | MINASARI |  |  | x |  |  |  | No |  |  |  | Web of Science |
| 56 | CHEON |  |  |  |  | x |  | No |  | Nigella damascena oil other active priciple |  | Web of Science |
| 57 | SINGH |  |  |  |  | x |  | No |  | mixture |  | Web of Science |
| 58 | CHUYEON |  |  |  |  | x |  | No |  | indication: bad breath |  | Web of Science |
| 59 | ISHIKAWA |  |  |  |  | x |  | No |  | mouthwash product, no clinical study |  | Web of Science |
| 60 | WALINSKI |  |  | x |  |  |  | No |  |  |  | Web of Science |
| 61 | [Not available] |  |  |  |  | x |  | No |  | summary |  | Web of Science |
| 62 | STEFANUS |  |  |  |  | x |  | No |  | summary |  | Web of Science |
| 63 | ALBERT |  |  |  |  | x |  | No |  | summary |  | Web of Science |
| 64 | MOHAMMAD |  |  |  |  | x |  | No |  | mixture |  | Web of Science |
| 65 |  |  |  |  |  |  |  |  |  |  | identical publication indexed in PubMed (Record 3) | Web of Science |
| 66 | Canakci |  | x |  |  |  |  | No |  |  |  | Web of Science |
| 67 | Egilmez |  | x |  |  |  |  | No |  |  |  | Web of Science |
| 68 | Mathur |  |  |  | x |  |  | No |  | metabolic disorders |  | Web of Science |
| 69 | Farrag |  | x |  |  |  |  | No |  |  |  | Web of Science |
| 70 |  |  |  |  |  |  |  |  |  |  | identical publication indexed in PubMed (Record 6) | Web of Science |
| 71 |  |  |  |  |  |  |  |  |  |  | identical publication indexed in PubMed (Record 8) | Web of Science |
| 72 |  |  |  |  |  |  |  |  |  |  | identical publication indexed in PubMed (Record 22) | Web of Science |
| 73 | ERNIE |  |  |  |  | x |  | No |  | summary |  | Web of Science |
| 74 | GUPTA |  |  |  |  | x |  | No |  | mixture |  | Web of Science |
| 75 | SETIAWATIE |  |  |  |  | x |  | No |  | summary |  | Web of Science |
| 76 | Xu |  |  | x |  |  |  | No |  |  |  | Web of Science |
| 77 |  |  |  |  |  |  |  |  |  | periodontitis | Duplicate of PubMed Record 36 | Web of Science |
| 78 | Al-Wafi |  |  |  |  | x |  | No |  | summary, caries |  | Web of Science |
| 79 |  |  |  |  |  |  |  |  |  |  | identical publication indexed in PubMed (Record 25) | Web of Science |
| 80 | SELA |  |  |  |  | x |  | No |  | summary |  | Web of Science |
| 81 |  |  |  |  |  |  |  |  |  | gingivitis | Duplicate of PubMed Record 33 | Web of Science |
| 82 |  |  |  |  |  |  |  |  |  |  | identical publication indexed in PubMed (Record 29) | Web of Science |
| 83 | Tawfig |  |  | x |  |  |  | No |  |  |  | Web of Science |
| 84 | Hasheminasab | x |  |  |  |  |  | No |  | mixture |  | Web of Science |
| 85 | [Not available] |  |  |  |  | x |  | No |  | mixture |  | Web of Science |
| 86 |  |  |  |  |  |  |  |  |  | gingivitis |  | Web of Science |
| 87 | Al-Thobity |  |  | x |  |  |  | No |  |  | identical publication indexed in PubMed (Record 21) | Web of Science |
| 88 | Yuslianti |  |  | x |  |  |  | No |  | mixture |  | Web of Science |
| 89 | Al-Bayaty | x |  |  |  |  |  | Yes |  | periodontitis |  | Web of Science |
| 90 |  |  |  |  |  |  |  |  |  | mixture |  | Web of Science |
| 91 | Albarrag |  |  | x |  |  |  | No |  |  |  | Web of Science |
| 92 |  |  |  |  |  |  |  |  |  |  | identical publication indexed in PubMed (Record 30) | Web of Science |
| 93 |  |  |  |  |  |  |  |  |  |  | identical publication indexed in PubMed (Record 24) | Web of Science |
| 94 | Kara |  | x |  |  |  |  | No |  |  |  | Web of Science |
| 95 |  |  |  |  |  |  |  |  |  |  | identical publication indexed in PubMed (Record 9) | Web of Science |
| 96 |  |  |  |  |  |  |  |  |  |  | identical publication indexed in PubMed (Record 11) | Web of Science |
| 97 |  |  |  |  |  |  |  |  |  |  | identical publication indexed in PubMed (Record 20) | Web of Science |
| 98 | SHIFA |  |  |  |  | x |  | No |  | summary |  | Web of Science |
| 99 | Zhao |  | x |  |  |  |  | No |  | suimvastatin |  | Web of Science |
| 100 | Tufan |  | x |  |  |  |  | No |  | whey protein |  | Web of Science |
| 101 | Kara, |  | x |  |  |  |  | No |  | Ginkgo |  | Web of Science |
| 102 | Aktas |  | x |  |  |  |  | No |  | misoprostol |  | Web of Science |
| 1 | IRCT20221212056786N2 | x |  |  |  |  | x | No |  | Trial registry record corresponding to Arab Farashahi et al.; not a separate report for inclusion. |  | Cochrane |
| 2 | Rahman | x |  |  |  |  | x | No |  | Gingivitis; trial completed, but no published results available. |  | Cochrane |
| 3 |  |  |  |  |  |  |  |  |  | Periodontitis | Duplicate of PubMed Record 36 | Cochrane |
| 4 | Elamrousy | x |  |  |  |  | x | No |  | Grade C periodontitis; excluded a priori because it is outside the predefined scope of the review. |  | Cochrane |
| 5 | CTRI/2021/04/033045, | x |  |  |  |  | x | No |  | Trial registry/protocol corresponding to Khan et al.; not a separate report for inclusion. |  | Cochrane |
| 6 | IRCT2016021826637N1, | x |  |  |  |  | x | No | x | Clinical trial registry record on chronic periodontitis; results/publication not identified. |  | Cochrane |
| 7 | NCT05497895, | x |  |  |  |  | x | No |  | Trial registry/protocol corresponding to Almehmadi et al.; not a separate report for inclusion. |  | Cochrane |
| 8 | CTRI/2026/02/103911, | x |  |  |  | x |  | No |  | Clinical trial outside the review scope: root-canal irrigation in apical periodontitis. |  | Cochrane |
| 9 | NCT05693597, | x |  |  |  | x |  | No |  | Clinical trial outside the review scope: radiation-induced dermatitis. |  | Cochrane |
| 10 |  |  |  |  |  |  |  |  |  | Gingivitis | Duplicate of PubMed Record 33 | Cochrane |
| 11 | IRCT2016100914106N5, | x |  |  |  |  | x | No |  | Clinical trial outside the review intervention: the registry details describe pomegranate gel rather than TQ. |  | Cochrane |
| 12 | CTRI/2021/12/038575, | x |  |  |  |  | x | No | x | Relevant oral-mucositis trial registry record (0.1% TQ gel); results/publication not identified. Not the Ameen oil-mouthwash study. |  | Cochrane |
| 13 |  |  |  |  |  |  |  |  |  | Mucositis | identical publication indexed in PubMed (Record 10) | Cochrane |
| 14 |  |  |  |  |  |  |  |  |  | Registry record corresponding to the published study. | Registry record corresponding to PubMed (Record 33) | Cochrane |
| 15 | CTRI/2025/10/095677, | x |  |  |  | x |  | No |  | Periodontitis C was excluded from our SR |  | Cochrane |
| 16 |  |  |  |  |  |  |  |  |  | Mucositis | identical publication indexed inEmbase (Record 50) | Cochrane |
| 17 | IRCT20220516054874N21, | x |  |  |  |  | x | No | x | Potentially relevant gingivitis trial using Nigella sativa chewing gum; results/publication not identified. |  | Cochrane |
| 18 | IRCT20200720048145N2, | x |  |  |  | x |  | No |  | Clinical trial outside the review scope: vulvovaginal mucositis. |  | Cochrane |
| 19 | NCT06013202, | x |  |  |  | x |  | No |  | Clinical trial outside the predefined disease scope: recurrent minor aphthous ulcer. |  | Cochrane |
| 20 | IRCT20190406043175N13, | x |  |  |  |  | x | No |  | Multi-ingredient toothpaste; the specific effect of Nigella sativa cannot be isolated. |  | Cochrane |
| 21 | NCT04722380, | x |  |  |  | x |  | No |  | Periodontitis C was excluded from our SR |  | Cochrane |
| 22 |  |  |  |  |  |  |  |  |  | Gingivitis | identical publication indexed in PubMed (Record 21) | Cochrane |
| 23 | NCT05069246, | x |  |  |  |  | x | No |  | Trial registry record corresponding to Rahman et al.; not a separate report for inclusion. |  | Cochrane |
| 24 | CTRI/2025/01/079131, | x |  |  |  | x |  | No |  | Periodontitis C was excluded from our SR |  | Cochrane |
| 25 | CTRI/2024/01/061413, | x |  |  |  |  | x | No |  | Polyherbal mouthwash; the specific effect of Nigella sativa cannot be isolated. |  | Cochrane |

**Supplementary Table S2b. Study identification and classification of records retrieved from the five databases after deduplication**

| **No.** | **Study** | **Clinical** | **Animal** | **In vitro** | **Review** | **Other** | **Potentially eligible** | **Included in review** | **Notes** | **Database** |
| --- | --- | --- | --- | --- | --- | --- | --- | --- | --- | --- |
| PubMed | | | | | | | | | | |
| 1 | Mekhemar |  |  |  | x |  | No |  |  |  |
| 2 | Al-Attass |  |  |  | x |  | No |  |  |  |
| 3 | Tada |  |  | x |  |  | No |  |  |  |
| 4 | Madani |  | x |  |  |  | No |  |  |  |
| 5 | Singh | x |  |  |  |  | Yes | 1 | Gingivitis |  |
| 6 | Setiawatie |  | x |  |  |  | No |  |  |  |
| 7 | Tawfig, |  |  | x |  |  | No |  |  |  |
| 8 | Alicea |  |  |  | x |  | No |  |  |  |
| 9 | Tantivitayakul |  |  | x |  |  | No |  |  |  |
| 10 | Hussain | x |  |  |  |  | Yes | 1 | Mucositis |  |
| 11 | Kaypetch |  |  | x |  |  | No |  |  |  |
| 12 | Bhavikatti |  |  | x |  |  | No |  |  |  |
| 13 | Varela-López |  |  |  | x |  | No |  |  |  |
| 14 | Khan |  |  |  | x |  | No |  |  |  |
| 15 | Sultan |  |  | x |  |  | No |  |  |  |
| 16 | Ozdemir |  | x |  |  |  | No |  |  |  |
| 17 | Afşar |  | x |  |  |  | No |  |  |  |
| 18 | Nazemi Salman |  |  | x |  |  | No |  |  |  |
| 19 | Maduratna |  | x |  |  |  | No |  |  |  |
| 20 | Huang |  |  |  | x |  | No |  |  |  |
| 21 | Arab Farashahi | x |  |  |  |  | Yes | 1 | Gingivitis |  |
| 22 | Sthanislas |  |  | x |  |  | No |  |  |  |
| 23 | Eğilmez |  | x |  |  |  | No |  |  |  |
| 24 | Thummuri |  |  | x |  |  | No |  |  |  |
| 25 | Ariamanesh |  |  | x |  |  | No |  |  |  |
| 26 | Vlachojannis |  |  | x |  |  | No |  |  |  |
| 27 | Laskar |  |  | x |  |  | No |  |  |  |
| 28 | Kiari |  |  | x |  |  | No |  |  |  |
| 29 | Acipinar |  | x |  |  |  | No |  |  |  |
| 30 | Maraghehpour |  |  | x |  |  | No |  |  |  |
| 31 | Al-Khalifa |  |  | x |  |  | No |  |  |  |
| 32 | Tariq |  |  |  |  | x | No |  | mixture |  |
| 33 | Almehmadi | x |  |  |  |  | Yes |  | Gingivitis |  |
| 34 | Xu |  |  | x |  |  | No |  |  |  |
| 35 | Çanakci |  | x |  |  |  | No |  |  |  |
| 36 | Kapil | x |  |  |  |  | Yes |  | Periodontitis |  |
| 37 | Al-Thobity |  | x |  |  |  | No |  |  |  |
| 38 | Konstantinidis, |  |  |  | x |  | No |  |  |  |
|  | Summary | 5 | 9 | 16 | 7 | 1 |  |  |  |  |
| Embase | | | | | | | | | | |
| 1 | Alicea |  |  |  | x |  | No |  |  |  |
| 2 | Ganeshkumar |  |  | x |  |  | No |  |  |  |
| 3 | Anonymous | x |  |  |  |  | No |  | Results not available in VClinicalTrials.gov |  |
| 4 | Anonymous | x |  |  |  |  | No |  | Protocol |  |
| 5 | Anonymous | x |  |  |  |  | No |  | Radiation-induced Dermatitis |  |
| 6 | Anonymous |  |  |  |  | x | No |  | aggressive periodontitis |  |
| 7 | Anonymous | x |  |  |  |  | No |  | registry record corresponding to Rahman et al. 2023 |  |
| 8 | Anonymous | x |  |  |  |  | No |  | registry record; duplicate of the Almehmadi publication. |  |
| 9 | Anonymous |  |  |  |  | x | No |  | no clinical study |  |
| 10 | Anonymous | x |  |  |  |  | No |  | Gastritis |  |
| 11 | Khan |  |  | x |  |  | No |  |  |  |
| 12 |  |  |  |  |  |  |  |  |  | identical publication indexed in PubMed (Record 29) |
| 13 | Afsar |  | x |  |  |  | No |  |  |  |
| 14 |  |  |  |  |  |  |  |  | Gingivitis | identical publication indexed in PubMed (Record 21) |
| 15 | Thanasekaran | x |  |  |  |  | Yes |  | Peri-implantitis |  |
| 16 | Bhavikatti |  |  | x |  |  | No |  |  |  |
| 17 | Tungare |  |  |  | x |  | No |  |  |  |
| 18 | Ghasemi |  |  |  | x |  | No |  |  |  |
| 19 | Ahmad |  |  |  | x |  | No |  |  |  |
| 20 | Bhavikatti |  |  |  | x |  | No |  |  |  |
| 21 | Joshi |  |  |  | x |  | No |  |  |  |
| 22 | Belgaumi |  |  | x |  |  | No |  |  |  |
| 23 | Madani |  | x |  |  |  | No |  |  |  |
| 24 | Bakhtiari |  | x |  |  |  | No |  |  |  |
| 25 | Tawfig |  |  | x |  |  | No |  |  |  |
| 26 | Rahman | x |  |  |  |  | No |  | no results available - ClinicalTrials.gov |  |
| 27 | Qutieshat |  |  |  | x |  | No |  |  |  |
| 28 | Orororo |  | x |  |  |  | No |  |  |  |
| 29 | Ulfah |  |  | x |  |  | No |  |  |  |
| 30 | Lima |  |  |  | x |  | No |  |  |  |
| 31 | Dilber |  | x |  |  |  | No |  |  |  |
| 32 | Oliveira |  |  | x |  |  | No |  |  |  |
| 33 | Servi |  |  | x |  |  | No |  |  |  |
| 34 | Marmitt |  |  |  | x |  | No |  |  |  |
| 35 | Kazemi |  | x |  |  |  | No |  |  |  |
| 36 | Ahuja |  |  |  | x |  | No |  |  |  |
| 37 | Sultan |  |  | x |  |  | No |  |  |  |
| 38 |  |  |  |  |  |  |  |  |  | identical publication indexed in PubMed (Record 6) |
| 39 | Asri |  |  |  | x |  | No |  |  |  |
| 40 |  |  |  |  |  |  |  |  |  | identical publication indexed in PubMed (Record 31) |
| 41 | Kurnia |  |  | x |  |  | No |  |  |  |
| 42 | Xu |  |  | x |  |  | No |  |  |  |
| 43 |  |  |  |  |  |  |  |  |  | identical publication indexed in PubMed (Record 3) |
| 44 | Tantivitayakul |  |  | x |  |  | No |  |  | identical publication indexed in PubMed (Record 9) |
| 45 | Alwafi |  |  |  |  | x | No |  | Epidemiological study |  |
| 46 | Tsukatani |  |  | x |  |  | No |  |  |  |
| 47 | Anonymous |  |  |  |  | x | No |  | Ni clinical study |  |
| 48 | Alhiyari |  | x |  |  |  | No |  |  |  |
| 49 |  |  |  |  |  |  |  |  | Mucositis | identical publication indexed in PubMed (Record 10) |
| 50 | Ameen | x |  |  |  |  | Yes |  | Mucositis |  |
| 51 | Cheraghi |  |  |  | x |  | No |  |  |  |
| 52 |  |  |  |  |  |  |  |  | Periodontitis | Duplicate of PubMed Record 36 |
| 53 |  |  |  |  |  |  |  |  |  | identical publication indexed in PubMed (Record 26) |
| 54 | Moayer |  | x |  |  |  | No |  |  |  |
| 55 | Elamrousy | x |  |  |  |  | No |  | No full-text retrieval was pursued because studies on Grade C periodontitis were excluded a priori according to the predefined eligibility criteria. |  |
| 56 | Canakci |  | x |  |  |  | No |  |  |  |
| 57 |  |  |  |  |  |  |  |  |  | identical publication indexed in PubMed (Record 27) |
| 58 | Al-Thobity |  |  | x |  |  | No |  |  |  |
| 59 | Naeini |  |  | x |  |  | No |  |  |  |
| 60 | El-Sheikh |  | x |  |  |  | No |  |  |  |
| 61 | Uzun |  |  |  |  |  | No |  | Epidemiological study |  |
| 62 |  |  |  |  |  |  |  |  |  | identical publication indexed in PubMed (Record 2) |
| 63 | Mahdizadeh |  |  |  | x |  | No |  |  |  |
| 64 | Kafar |  |  |  |  |  | No |  | Epidemiological study |  |
| 65 | Guler |  |  |  | x |  | No |  |  |  |
| 66 |  |  |  |  |  |  |  |  |  | identical publication indexed in PubMed (Record 24) |
| 67 | Farrag |  | x |  |  |  | No |  |  |  |
| 68 | Osman |  |  |  |  |  | No |  | Coeliac Disease, Case Report |  |
| 69 |  |  |  |  |  |  |  |  |  | identical publication indexed in PubMed (Record 16) |
| 70 | Kokoska |  |  | x |  |  | No |  |  |  |
|  | Summary | 13 | 15 | 19 | 16 | 4 |  |  |  |  |
| Scopus | | | | | | | | | | |
| 1 |  |  |  |  |  |  |  |  |  | identical publication indexed in PubMed (Record 2) |
| 2 | Bhavikatti |  |  | x |  |  | No |  |  |  |
|  | Summary |  |  | 1 | 1 |  |  |  |  |  |
| Web of Science | | | | | | | | | | |
| 1 | Kokoska, |  |  | x |  |  | No |  |  |  |
| 2 |  |  |  |  |  |  |  |  |  | identical publication indexed in PubMed (Record 1) |
| 3 | Afsar |  | x |  |  |  | No |  |  |  |
| 4 |  |  |  |  |  |  |  |  |  | identical publication indexed in PubMed (Record 16) |
| 5 |  |  |  |  |  |  |  |  |  | identical publication indexed in PubMed (Record 18) |
| 6 | Moayer |  | x |  |  |  | No |  |  |  |
| 7 | Varela-Lopez |  |  |  | x |  | No |  |  |  |
| 8 | Supandi |  | x |  |  |  | No |  |  |  |
| 9 | Sultan |  |  | x |  |  | No |  |  |  |
| 10 |  |  |  |  |  |  |  |  |  | identical publication indexed in PubMed (Record 2) |
| 11 | Anonymous, Khan |  |  |  |  | x | No |  | mixed product |  |
| 12 | Alhiyari |  |  |  |  | x | No |  | Xerostoma |  |
| 13 |  |  |  |  |  |  |  |  |  | identical publication indexed in PubMed (Record 26) |
| 14 |  |  |  |  |  |  |  |  |  | identical publication indexed in PubMed (Record 19) |
| 15 | Alamoudi |  |  | x |  |  | No |  |  |  |
| 16 | Rahman | x |  |  |  |  | No |  | Gingivitis |  |
| 17 | Kang |  |  | x |  |  | No |  |  |  |
| 18 |  |  |  |  |  |  |  |  |  | identical publication indexed in PubMed (Record 31) |
| 19 | Al-Wafi |  | x |  |  |  | No |  |  |  |
| 20 | Khan |  |  |  | x |  | No |  |  |  |
| 21 | Dirjomuljono |  |  |  |  |  | No |  | other plant |  |
| 22 | Dilber |  | x |  |  |  | No |  |  |  |
| 23 | Ren |  |  | x |  |  | No |  |  |  |
| 24 | Anonymous |  |  | x |  |  | No |  |  |  |
| 25 |  |  |  |  |  |  |  |  |  | identical publication indexed in PubMed (Record 28) |
| 26 | [Anonymous] |  |  | x |  |  | No |  |  |  |
| 27 | Srinivasan |  |  |  | x |  | No |  |  |  |
| 28 | Tabassum |  |  |  | x |  | No |  |  |  |
| 29 | Kazemi |  | x |  |  |  | No |  |  |  |
| 30 | Kurnia |  |  |  | x |  | No |  |  |  |
| 31 | Zhang |  |  | x |  |  | No |  | Rosmarinic acid |  |
| 32 |  |  |  |  |  |  |  |  | Gingivitis | identical publication indexed in PubMed (Record 5) |
| 33 | Abdelfadil |  |  | x |  |  | No |  |  |  |
| 34 | Rajkamal |  | x |  |  |  | No |  |  |  |
| 35 | Hussein |  |  | x |  |  | No |  |  |  |
| 36 | Alaufi |  |  | x |  |  | No |  |  |  |
| 37 | Tucakov |  |  | x |  |  | No |  |  |  |
| 38 | Sallal, |  |  | x |  |  | No |  |  |  |
| 39 | Sanpinit |  | x |  |  |  | No |  |  |  |
| 40 | Fath |  |  | x |  |  | No |  |  |  |
| 41 | Madani |  | x |  |  |  | No |  |  |  |
| 42 | Jo |  |  | x |  |  | No |  |  |  |
| 43 | Erdogan |  | x |  |  |  | No |  | other plant |  |
| 44 | Imran |  |  |  | x |  | No |  |  |  |
| 45 | Pu |  | x |  |  |  | No |  |  |  |
| 46 | Khan |  |  |  | x |  | No |  | dry sockets after tooth extraction |  |
| 47 |  |  |  |  |  |  |  |  | Mucositis | identical publication indexed in PubMed (Record 10) |
| 48 | Siriwattanasatorn |  |  | x |  |  | No |  |  |  |
| 49 | Pipalia | x |  |  |  |  | No |  | submucous fibrosis |  |
| 50 | Besra |  |  | x |  |  | No |  |  |  |
| 51 | Dirjomuljono | x |  |  |  |  | No |  | mixture |  |
| 52 | Bhavikatti |  |  | x |  |  | No |  |  |  |
| 53 | Almatroodi |  |  |  | x |  | No |  | cancer |  |
| 54 |  |  |  |  |  |  |  |  |  | identical publication indexed in PubMed (Record 27) |
| 55 | MINASARI |  |  | x |  |  | No |  |  |  |
| 56 | CHEON |  |  |  |  | x | No |  | Nigella damascena oil other active priciple |  |
| 57 | SINGH |  |  |  |  | x | No |  | mixture |  |
| 58 | CHUYEON |  |  |  |  | x | No |  | indication: bad breath |  |
| 59 | ISHIKAWA |  |  |  |  | x | No |  | mouthwash product, no clinical study |  |
| 60 | WALINSKI |  |  | x |  |  | No |  |  |  |
| 61 | [Not available] |  |  |  |  | x | No |  | summary |  |
| 62 | STEFANUS |  |  |  |  | x | No |  | summary |  |
| 63 | ALBERT |  |  |  |  | x | No |  | summary |  |
| 64 | MOHAMMAD |  |  |  |  | x | No |  | mixture |  |
| 65 |  |  |  |  |  |  |  |  |  | identical publication indexed in PubMed (Record 3) |
| 66 | Canakci |  | x |  |  |  | No |  |  |  |
| 67 | Egilmez |  | x |  |  |  | No |  |  |  |
| 68 | Mathur |  |  |  | x |  | No |  | metabolic disorders |  |
| 69 | Farrag |  | x |  |  |  | No |  |  |  |
| 70 |  |  |  |  |  |  |  |  |  | identical publication indexed in PubMed (Record 6) |
| 71 |  |  |  |  |  |  |  |  |  | identical publication indexed in PubMed (Record 8) |
| 72 |  |  |  |  |  |  |  |  |  | identical publication indexed in PubMed (Record 22) |
| 73 | ERNIE |  |  |  |  | x | No |  | summary |  |
| 74 | GUPTA |  |  |  |  | x | No |  | mixture |  |
| 75 | SETIAWATIE |  |  |  |  | x | No |  | summary |  |
| 76 | Xu |  |  | x |  |  | No |  |  |  |
| 77 |  |  |  |  |  |  |  |  | periodontitis | Duplicate of PubMed Record 36 |
| 78 | Al-Wafi |  |  |  |  | x | No |  | summary, caries |  |
| 79 |  |  |  |  |  |  |  |  |  | identical publication indexed in PubMed (Record 25) |
| 80 | SELA |  |  |  |  | x | No |  | summary |  |
| 81 |  |  |  |  |  |  |  |  | gingivitis | Duplicate of PubMed Record 33 |
| 82 |  |  |  |  |  |  |  |  |  | identical publication indexed in PubMed (Record 29) |
| 83 | Tawfig |  |  | x |  |  | No |  |  |  |
| 84 | Hasheminasab | x |  |  |  |  | No |  | mixture |  |
| 85 | [Not available] |  |  |  |  | x | No |  | mixture |  |
| 86 |  |  |  |  |  |  |  |  | gingivitis |  |
| 87 | Al-Thobity |  |  | x |  |  | No |  |  | identical publication indexed in PubMed (Record 21) |
| 88 | Yuslianti |  |  | x |  |  | No |  | mixture |  |
| 89 | Al-Bayaty | x |  |  |  |  | Yes |  | periodontitis |  |
| 90 |  |  |  |  |  |  |  |  | mixture |  |
| 91 | Albarrag |  |  | x |  |  | No |  |  |  |
| 92 |  |  |  |  |  |  |  |  |  | identical publication indexed in PubMed (Record 30) |
| 93 |  |  |  |  |  |  |  |  |  | identical publication indexed in PubMed (Record 24) |
| 94 | Kara |  | x |  |  |  | No |  |  |  |
| 95 |  |  |  |  |  |  |  |  |  | identical publication indexed in PubMed (Record 9) |
| 96 |  |  |  |  |  |  |  |  |  | identical publication indexed in PubMed (Record 11) |
| 97 |  |  |  |  |  |  |  |  |  | identical publication indexed in PubMed (Record 20) |
| 98 | SHIFA |  |  |  |  | x | No |  | summary |  |
| 99 | Zhao |  | x |  |  |  | No |  | suimvastatin |  |
| 100 | Tufan |  | x |  |  |  | No |  | whey protein |  |
| 101 | Kara, |  | x |  |  |  | No |  | Ginkgo |  |
| 102 | Aktas |  | x |  |  |  | No |  | misoprostol |  |
|  | Summary | 10 | 24 | 37 | 13 | 18 |  |  |  |  |
| Cochrane | | | | | | | | | | |
| 1 | IRCT20221212056786N2 | x |  |  |  |  | No |  | Trial registry record corresponding to Arab Farashahi et al.; not a separate report for inclusion. |  |
| 2 | Rahman | x |  |  |  |  | No |  | Gingivitis; trial completed, but no published results available. |  |
| 3 |  |  |  |  |  |  |  |  | Periodontitis | Duplicate of PubMed Record 36 |
| 4 | Elamrousy | x |  |  |  |  | No |  | Grade C periodontitis; excluded a priori because it is outside the predefined scope of the review. |  |
| 5 | CTRI/2021/04/033045, | x |  |  |  |  | No |  | Trial registry/protocol corresponding to Khan et al.; not a separate report for inclusion. |  |
| 6 | IRCT2016021826637N1, | x |  |  |  |  | No |  | Clinical trial registry record on chronic periodontitis; results/publication not identified. |  |
| 7 | NCT05497895, | x |  |  |  |  | No |  | Trial registry/protocol corresponding to Almehmadi et al.; not a separate report for inclusion. |  |
| 8 | CTRI/2026/02/103911, | x |  |  |  | x | No |  | Clinical trial outside the review scope: root-canal irrigation in apical periodontitis. |  |
| 9 | NCT05693597, | x |  |  |  | x | No |  | Clinical trial outside the review scope: radiation-induced dermatitis. |  |
| 10 |  |  |  |  |  |  |  |  | Gingivitis | Duplicate of PubMed Record 33 |
| 11 | IRCT2016100914106N5, | x |  |  |  |  | No |  | Clinical trial outside the review intervention: the registry details describe pomegranate gel rather than TQ. |  |
| 12 | CTRI/2021/12/038575, | x |  |  |  |  | No |  | Relevant oral-mucositis trial registry record (0.1% TQ gel); results/publication not identified. Not the Ameen oil-mouthwash study. |  |
| 13 |  |  |  |  |  |  |  |  | Mucositis | identical publication indexed in PubMed (Record 10) |
| 14 |  |  |  |  |  |  |  |  | Registry record corresponding to the published study. | Registry record corresponding to PubMed (Record 33) |
| 15 | CTRI/2025/10/095677, | x |  |  |  | x | No |  | Periodontitis C was excluded from our SR |  |
| 16 |  |  |  |  |  |  |  |  | Mucositis | identical publication indexed inEmbase (Record 50) |
| 17 | IRCT20220516054874N21, | x |  |  |  |  | No |  | Potentially relevant gingivitis trial using Nigella sativa chewing gum; results/publication not identified. |  |
| 18 | IRCT20200720048145N2, | x |  |  |  | x | No |  | Clinical trial outside the review scope: vulvovaginal mucositis. |  |
| 19 | NCT06013202, | x |  |  |  | x | No |  | Clinical trial outside the predefined disease scope: recurrent minor aphthous ulcer. |  |
| 20 | IRCT20190406043175N13, | x |  |  |  |  | No |  | Multi-ingredient toothpaste; the specific effect of Nigella sativa cannot be isolated. |  |
| 21 | NCT04722380, | x |  |  |  | x | No |  | Periodontitis C was excluded from our SR |  |
| 22 |  |  |  |  |  |  |  |  | Gingivitis | identical publication indexed in PubMed (Record 21) |
| 23 | NCT05069246, | x |  |  |  |  | No |  | Trial registry record corresponding to Rahman et al.; not a separate report for inclusion. |  |
| 24 | CTRI/2025/01/079131, | x |  |  |  | x | No |  | Periodontitis C was excluded from our SR |  |
| 25 | CTRI/2024/01/061413, | x |  |  |  |  | No |  | Polyherbal mouthwash; the specific effect of Nigella sativa cannot be isolated. |  |
|  | Summary | 25 |  |  |  | 7 | 5 |  |  |  |

**Supplementary Table S3: Clinical studies included in the qualitative synthesis according to disease category and database source.**

| **Study** | **Gingivitis** | **Periodontitis** | **Mucositis** | **Peri-implantitis** | **Source database** |
| --- | --- | --- | --- | --- | --- |
| Singh | x |  |  |  | PubMed |
| Hussain |  |  | x |  | PubMed |
| Arab Farashahi | x |  |  |  | PubMed |
| Almehmadi | x |  |  |  | PubMed |
| Kapil |  | x |  |  | PubMed |
| Arab Farashahi |  |  |  |  | Embase |
| Thanasekaran |  |  |  |  | Embase |
| Hussain |  |  |  |  | Embase |
| Ameen |  |  |  |  | Embase |
| Kapil |  |  |  |  | Embase |
| Al-Bayaty |  | x |  |  | Web of Science |
| Hussain |  |  |  |  | Web of Science |
| Kapil |  |  |  |  | Web of Science |
| Almehmadi |  |  |  |  | Web of Science |
| Arab Farashahi |  |  |  |  | Web of Science |
| Al-Bayaty |  |  |  |  | Web of Science |
| Kapil |  |  |  |  | Cochrane |
| Almehmadi |  |  |  |  | Cochrane |
| Hussain |  |  |  |  | Cochrane |
| Arab Farashahi |  |  |  |  | Cochrane |
| Ameen |  |  |  |  | Cochrane |

**Supplementary Table S4. Risk of bias assessment of randomized clinical trials investigating Nigella sativa or thymoquinone in gingivitis, oral mucositis, periodontitis, and peri-implantitis using the Cochrane Risk of Bias 2 (RoB 2) tool.**

| Supplementary Table 3 |  |  |
| --- | --- | --- |
| Risk of bias assessment of randomized clinical trials investigating Nigella sativa or thymoquinone in gingivitis and oral mucositis using the Cochrane Risk of Bias 2 (RoB 2) tool. |  |  |
| RoB 2 = revised Cochrane Risk of Bias tool for randomized trials. |  |  |
| RoB 2 domain | Judgement | Justification |
| Gingivitis/Mucositis Studies |  |  |
| Arab Farashahi et al., 2026 |  |  |
| Bias arising from the randomization process | Low risk | Random sequence generation (Random Allocation Software), 1:1 randomization, allocation concealment using sequentially numbered opaque sealed envelopes, comparable baseline characteristics. |
| Bias due to deviations from intended interventions | Low risk | Triple-blind design (participants, outcome assessors, statisticians), identical opaque bottles, intention-to-treat analysis, high compliance monitoring, no protocol deviations reported. |
| Bias due to missing outcome data | Low risk | All 36 randomized participants completed the study; No missing outcome data were reported, and all randomized participants were included in the analysis. |
| Bias in measurement of the outcome | Low risk | Outcomes were assessed by the same trained blinded examiner using validated periodontal indices (PI, BI, SI). Outcome assessment was unlikely to be influenced by knowledge of the intervention. |
| Bias in selection of the reported result | Some concerns | The trial was prospectively registered and the primary outcome was prespecified. However, the study does not explicitly report a predefined statistical analysis plan finalized before unblinded data analysis, so ROB 2 recommends Some concerns rather than Low risk. |
| Singh et al., 2019 |  |  |
| Bias arising from the randomization process | Low risk | Computer-generated randomization with allocation concealment using opaque sealed envelopes was reported. No important baseline imbalances between intervention and control sites were identified. |
| Bias due to deviations from intended interventions | Some concerns | No information was provided regarding blinding of participants or personnel. However, no protocol deviations or deviations from the intended interventions were reported. |
| Bias due to missing outcome data | Low risk | No missing outcome data were reported, and all randomized participants were included in the analysis. |
| Bias in measurement of the outcome | Some concerns | Clinical outcomes (GI and PI) were assessed using established indices, but blinding of the outcome assessor was not reported. Therefore, detection bias cannot be excluded. |
| Bias in selection of the reported result | Some concerns | No study protocol or trial registration was available; therefore, selective outcome reporting cannot be ruled out. |
| Ilangovan 2021 |  |  |
| Bias arising from the randomization process | Low risk | Participants were randomly allocated using a simple lottery method by an independent person not involved in the study. Baseline plaque index (PI) and gingival index (GI) were comparable between the two groups, with no evidence of important baseline imbalances. |
| Bias due to deviations from intended interventions | Low risk | The trial was double-blinded. Participants and the outcome examiner were blinded to group allocation, and both mouthwashes were dispensed in identical bottles. No deviations from the intended interventions were reported. |
| Bias due to missing outcome data | Low risk | No missing outcome data were reported, and all randomized participants were included in the analysis. |
| Bias in measurement of the outcome | Low risk | Clinical outcomes (PI and GI) were assessed using validated periodontal indices by a blinded examiner, minimizing the risk of detection bias. |
| Bias in selection of the reported result | Some concerns | No prospective trial registration or published study protocol was reported. Consequently, selective reporting of outcomes cannot be excluded. |
| Khan et al., 2021 |  |  |
| Bias arising from the randomization process | Some concerns | Participants were reported to be randomly allocated to the intervention groups; however, the method used to generate the random sequence and allocation concealment were not described. Baseline characteristics were comparable between the groups. |
| Bias due to deviations from intended interventions | Low risk | The study was single-blinded because participants and their parents had to be informed about the allocated treatment. Outcome assessment was performed by an independent blinded examiner, and no deviations from the intended interventions or protocol violations were reported. |
| Bias due to missing outcome data | Low risk | One participant was lost to follow-up because of unwillingness to attend recall visits. The dropout rate was low (1/60, 1.7%) and unlikely to have influenced the study results. |
| Bias in measurement of the outcome | Low risk | Clinical outcomes were assessed by a calibrated independent examiner who was blinded to group allocation. Examiner calibration was reported (κ = 0.84), reducing the risk of detection bias. |
| Bias in selection of the reported result | Low risk | The trial was prospectively registered in the Clinical Trials Registry of India (CTRI/2021/04/033045), reducing the likelihood of selective outcome reporting. |
| Almehmadi et al., 2024 |  |  |
| Bias arising from the randomization process | Some concerns | Participants were allocated using stratified randomization with allocation concealment. However, the method used to generate the random sequence was not described in detail, and baseline demographic and clinical characteristics were not sufficiently reported to assess group comparability. |
| Bias due to deviations from intended interventions | Low risk | The study was double-blinded. Participants, investigators, and personnel assessing the clinical outcomes were blinded to treatment allocation. Treatment compliance was monitored using diary cards and drug accountability records, and no relevant protocol deviations were reported. |
| Bias due to missing outcome data | Low risk | Three participants (4.8%) were lost to follow-up because of non-compliance (one from each group). The dropout rate was low and balanced across groups and is unlikely to have affected the study results. |
| Bias in measurement of the outcome | Low risk | Clinical outcomes were assessed by two calibrated independent examiners using validated periodontal indices. Inter-examiner reliability was reported (κ = 0.67), and outcome assessors were blinded to treatment allocation. |
| Bias in selection of the reported result | Low risk | The study protocol was prospectively registered (ClinicalTrials.gov: NCT05497895), minimizing the risk of selective outcome reporting. |
| Hussain 2019 |  |  |
| Bias arising from the randomization process | Some concerns | Participants were randomly allocated using simple randomization with a 1:1 allocation ratio. Allocation concealment was ensured by personnel not involved in recruitment or outcome assessment. However, the method used to generate the random sequence was not described in sufficient detail. Baseline demographic and disease characteristics were comparable between the groups. |
| Bias due to deviations from intended interventions | Some concerns | The study was open-label. Neither participants nor treating personnel were blinded to the allocated intervention. Although no protocol deviations were reported, performance bias cannot be excluded. |
| Bias due to missing outcome data | Low risk | Two participants (one in each group) died during induction chemotherapy. These losses were balanced between groups and were unrelated to the intervention. Outcome data for the remaining participants were complete. |
| Bias in measurement of the outcome | Some concerns | Clinical outcomes (WHO, NCI and OMAS scores) include subjective components. The study does not report whether outcome assessors were blinded; therefore, detection bias cannot be excluded. |
| Bias in selection of the reported result | Some concerns | No trial registration or published study protocol was reported. Consequently, selective outcome reporting cannot be excluded. |
| Ameen 2019 |  |  |
| Bias arising from the randomization process | Low risk | Participants were allocated using block randomization. Baseline demographic, clinical and tumor characteristics were comprehensively reported and were comparable between groups. No evidence of baseline imbalance was observed. |
| Bias due to deviations from intended interventions | Some concerns | The study was conducted as an open-label trial. Participants and treating clinicians were aware of the allocated intervention. Although treatment compliance was reported as excellent and no protocol deviations were described, performance bias cannot be excluded. |
| Bias due to missing outcome data | Low risk | No missing outcome data were reported, and all randomized participants were included in the analysis. |
| Bias in measurement of the outcome | Some concerns | Oral mucositis was assessed using the RTOG grading scale, which includes subjective clinical judgement. The study does not report whether the outcome assessors were blinded; therefore, detection bias cannot be excluded. |
| Bias in selection of the reported result | Some concerns | No prospective trial registration or publicly available study protocol was reported. Consequently, selective reporting cannot be ruled out. |
| Peri-implantitis/Periodontitis Studies |  |  |
| Thanasekaran |  |  |
| 1. Bias arising from the randomization process | Some concerns | The authors stated that participants were randomly allocated to the two study groups (n = 20 each). However, no information was provided regarding the method used to generate the random sequence or whether allocation concealment was implemented. Baseline characteristics (age and sex) were comparable between groups (p > 0.05), suggesting that the randomization process was likely successful, but insufficiently reported. |
| 2. Bias due to deviations from intended interventions | Some concerns | All mechanical debridement procedures were performed by a single operator using a standardized protocol. However, neither participant nor operator blinding was reported. Because the test group received an additional subgingival application of 0.2% thymoquinone gel, blinding was unlikely. Therefore, deviations from the intended interventions cannot be completely excluded. |
| 3. Bias due to missing outcome data | Low risk | No missing outcome data were reported, and all randomized participants were included in the analysis. |
| 4. Bias in measurement of the outcome | Low risk | Clinical outcomes (PI, GI, PPD, and CAL) were assessed using standardized and validated measurement methods. All measurements were performed by a single examiner at baseline and after 3 months. Although examiner blinding was not explicitly reported, the outcomes were primarily objective clinical measurements, reducing the likelihood of measurement bias. |
| 5. Bias in selection of the reported result | Some concerns | No study protocol or trial registration was reported. Consequently, it is unclear whether all pre-specified outcomes and analyses were reported as planned, and selective reporting of results cannot be ruled out. |
| Overall Risk of Bias | Some concerns | Although the study employed standardized intervention and assessment procedures and reported complete follow-up data, important methodological details regarding randomization, allocation concealment, blinding, and protocol registration were not provided. Therefore, the overall risk of bias is judged as presenting some concerns according to the Cochrane RoB 2 tool. |
| Kapil 2018 |  |  |
| 1. Bias arising from the randomization process | Low risk / Some concerns / High risk | The study adequately/inadequately reported the randomization process and allocation concealment procedures. Baseline characteristics were (or were not) comparable between groups, indicating a (successful/uncertain) randomization process. |
| 2. Bias due to deviations from intended interventions | Low risk / Some concerns / High risk | The interventions were delivered according to the study protocol. However, insufficient information regarding participant and/or operator blinding was provided, and deviations from the intended interventions cannot be completely ruled out. |
| 3. Bias due to missing outcome data | Low risk / Some concerns / High risk | No (or only minimal) missing outcome data or losses to follow-up were reported. There is no indication that missing data could have influenced the study results. |
| 4. Bias in measurement of the outcome | Low risk / Some concerns / High risk | Outcomes were assessed using standardized and validated measurement methods. Although examiner blinding was (not) reported, the clinical outcomes were primarily objective measurements, reducing the likelihood of measurement bias. |
| 5. Bias in selection of the reported result | Low risk / Some concerns / High risk | No study protocol or trial registration was reported. Therefore, it is unclear whether all pre-specified outcomes and analyses were reported as planned, and selective reporting cannot be ruled out. |
| Overall Risk of Bias | Low risk / Some concerns / High risk | The overall risk of bias was judged according to the Cochrane RoB 2 tool, taking into account the adequacy of the randomization process, adherence to the intended interventions, completeness of outcome data, outcome assessment methods, and the risk of selective reporting. |
| Khalil 2019 |  |  |
| 1. Bias arising from the randomization process | High risk | not randomized, strictly the ROBINS-I formate should have been emplyed. However, it is the only non-randomized study |
| 2. Bias due to deviations from intended interventions | Some concerns | Both groups received the planned periodontal treatment according to the study protocol. However, because the study was not randomized, baseline differences between the intervention groups cannot be excluded, resulting in some concerns regarding bias due to deviations from the intended interventions |
| 3. Bias due to missing outcome data | Low risk | No missing outcome data were reported, and all participants included in the study were analysed. There was no evidence that missing data influenced the study results. |
| 4. Bias in measurement of the outcome | Low risk | Clinical periodontal parameters and biochemical markers were assessed using established and standardized methods. There was no indication that outcome assessment differed between the study groups. |
| 5. Bias in selection of the reported result | Some concerns | No prospective trial registration or published study protocol was identified. Therefore, it cannot be confirmed that all prespecified outcomes and analyses were reported as originally planned. |
| Overall Risk of Bias | High risk | Strictly speaking, the ROBINS-I tool would have been the more appropriate instrument because randomization procedures were not reported. However, for consistency across all included studies, the study was additionally assessed within the RoB 2 framework. The resulting judgement indicates a high risk of bias. |
| Alaaeldin 2017 |  |  |
| 1. Bias arising from the randomization process | High risk | Randomization was reported; however, the method used to generate the random sequence and details on allocation concealment were insufficiently described, making it difficult to fully assess whether selection bias was adequately prevented. |
| 2. Bias due to deviations from intended interventions | Low risk | The intervention and control groups received the intended treatments according to the study protocol. There was no evidence of important deviations from the assigned interventions that were likely to influence the study outcomes. |
| 3. Bias due to missing outcome data | Low risk |  |
| 4. Bias in measurement of the outcome | Some concerns | Clinical outcome measurements were performed using standard periodontal parameters. However, blinding of the outcome assessor was not clearly reported, leaving some uncertainty regarding detection bias. |
| 5. Bias in selection of the reported result | Some concerns | No trial registration or published study protocol was identified. Therefore, it cannot be confirmed that all prespecified outcomes and analyses were reported. |
| Overall Risk of Bias | High risk | Strictly speaking, the ROBINS-I tool would have been the more appropriate instrument because randomization procedures were not reported. However, for consistency across all included studies, the study was additionally assessed within the RoB 2 framework. The resulting judgement indicates a high risk of bias. |
| Hassan 2020 |  |  |
| 1. Bias arising from the randomization process | Low risk | Participants were randomly assigned using a random number table. Allocation concealment was ensured by sequentially numbered identical bottles, and baseline characteristics were comparable between groups. bias was adequately prevented. |
| 2. Bias due to deviations from intended interventions | Low risk | The study was triple-blinded (participants, treating periodontist and data analyst). Both groups received identical non-surgical periodontal therapy and followed the intervention protocol without evidence of important deviations. study outcomes. |
| 3. Bias due to missing outcome data | Low risk | Although 20% of participants were lost to follow-up, the anticipated attrition had been incorporated into the sample size calculation, losses were balanced between groups, and there was no evidence that missing data affected the study outcomes. |
| 4. Bias in measurement of the outcome | Low risk | Salivary MMP-8 concentrations were determined using a standardized ELISA assay. Outcome assessment was objective, and blinding of investigators minimized the risk of measurement bias. |
| 5. Bias in selection of the reported result | Low risk | The trial was prospectively registered (ClinicalTrials.gov: NCT03270280), the primary outcome was predefined, and there was no evidence of selective reporting. |
| Overall Risk of Bias | Low risk | The overall risk of bias was judged as low because the study employed an appropriate randomization procedure with allocation concealment, triple blinding, prospective trial registration, predefined outcome measures and an a priori sample size calculation. Although 20% of participants were lost to follow-up, attrition had been anticipated during study planning, losses were balanced between groups, and there was no evidence that missing data or selective reporting influenced the results. |
| Al-Bayaty 2013 |  |  |
| 1. Bias arising from the randomization process | Low risk | The periodontal pockets were randomized into the three treatment groups before baseline assessment. Baseline measurements were obtained using standardized methods, and no evidence of baseline imbalance was reported. |
| 2. Bias due to deviations from intended interventions | Low risk | The study employed a single-blind design, and all interventions were performed according to a standardized protocol. Participants received identical oral hygiene instructions, and no deviations from the intended interventions were reported. |
| 3. Bias due to missing outcome data | Low risk |  |
| 4. Bias in measurement of the outcome | Low risk | Clinical outcomes were assessed by a single calibrated examiner who was blinded to the treatment allocation. Standardized and validated measurement methods were used, and intraexaminer agreement was excellent (κ = 0.81), minimizing the risk of measurement bias. |
| 5. Bias in selection of the reported result | Some concerns | No study protocol or trial registration was reported. Therefore, it cannot be confirmed whether all pre-specified outcomes and analyses were reported as planned. |
| Overall Risk of Bias | Low risk | The study demonstrated adequate randomization, blinded outcome assessment, complete follow-up, and standardized intervention procedures. Although no trial registration or published study protocol was reported, the overall risk of bias was judged to be low according to the Cochrane RoB 2 tool. |
| Nedumaran (2026) |  |  |
| 1. Bias arising from the randomization process | High risk | not randomized, strictly the ROBINS-I formate should have been emplyed. However, it is the only non-randomized study. The overall judgment of ROBINS-I is moderate risk) |
| 2. Bias due to deviations from intended interventions | Some concerns | The study followed a predefined intervention protocol, and there was no evidence of major deviations from the intended interventions. However, the non-randomized design increases the possibility of differences between groups that cannot be excluded. |
| 3. Bias due to missing outcome data | Low risk | No missing outcome data were reported, and all randomized participants were included in the analysis. |
| 4. Bias in measurement of the outcome | Low risk | Clinical outcomes were assessed using established periodontal parameters by standardized methods. There was no evidence that outcome measurement differed between the study groups. |
| 5. Bias in selection of the reported result | Some concerns | No prospective trial registration or published study protocol was identified. Therefore, it cannot be confirmed that all prespecified outcomes and analyses were reported. |
| Overall Risk of Bias | High risk | Strictly speaking, the ROBINS-I tool would have been the more appropriate instrument because the study was not randomized. However, to ensure consistency across all included studies, it was additionally assessed using the RoB 2 framework. Owing to the absence of randomization and the resulting high risk of bias in Domain 1, the overall RoB 2 judgement was high risk. According to the ROBINS-I tool, however, the study would have been classified as having a moderate risk of bias. |

**Supplementary Table S5. Narrative justification for the domain-specific Risk of Bias 2 (RoB 2) judgements.**

| **Study** | **Overall RoB 2 judgement** | **Narrative justification** |
| --- | --- | --- |
| Arab Farashahi et al., 2026 | Some concerns | The study was a well-conducted triple-blind randomized controlled trial with appropriate allocation concealment, complete follow-up, and blinded outcome assessment. Some concerns remained because no predefined statistical analysis plan finalized before unblinded data analysis was reported. |
| Singh et al., 2019 | Some concerns | Appropriate randomization and complete follow-up were reported. However, blinding of participants and outcome assessors was not described, and no trial registration or published protocol was available. |
| Ilangovan et al., 2021 | Some concerns | Randomization, double blinding and complete outcome data were adequately reported. The only remaining concern was the absence of prospective trial registration or a published study protocol. |
| Khan et al., 2021 | Low risk | Outcome assessment was blinded, follow-up was almost complete, and the trial was prospectively registered. Overall, the risk of bias was considered low. |
| Almehmadi et al., 2024 | Low risk | Stratified randomization, double blinding, calibrated outcome assessment and prospective trial registration supported a low overall risk of bias. |
| Hussain et al., 2019 | Some concerns | Open-label design, lack of assessor blinding and the absence of trial registration resulted in some concerns, although outcome data were complete. |
| Ameen et al., 2019 | Some concerns | Block randomization and complete follow-up were strengths. The open-label design and lack of prospective trial registration resulted in some concerns. |
| Thanasekaran et al., 2025 | Some concerns | Standardized interventions and complete follow-up were reported, but insufficient reporting of randomization, allocation concealment, blinding and protocol registration prevented a low-risk judgement. |
| Kapil et al., 2018 | Some concerns | The study was randomized and relied mainly on objective periodontal measurements. Insufficient information on blinding and absence of trial registration resulted in some concerns. |
| Khalil et al., 2019 | High risk | Randomization was not reported. Although ROBINS-I would have been more appropriate, RoB 2 was applied for consistency, resulting in an overall judgement of high risk. |
| Hassan et al., 2020 | Low risk | Triple blinding, allocation concealment, prospective trial registration and objective outcome assessment supported a low overall risk of bias. |
| Al-Bayaty et al., 2013 | Low risk | Adequate randomization, blinded outcome assessment, standardized measurements and complete follow-up supported a low overall risk of bias. |
| Nedumaran et al., 2026 | High risk | This non-randomized study would have been more appropriately assessed using ROBINS-I. RoB 2 was additionally applied for consistency, resulting in a high-risk judgement. |
